# The use of growth factors to modulate the activities of antigen–specific CD8+ T cells in vitro

**Published:** 2011-11-24

**Authors:** FQ Alenzi, FA Alenazi, Y Al-Kaabi, ML Salem

**Affiliations:** * College of Applied Medical Sciences, Prince Salman University, Saudi Arabia; ** Dept of Ped, KFMC, Riyadh, Saudi Arabia; *** College of Applied Medical Sciences, Dammam University, Saudi Arabia; **** Zoology Dept, Faculty of Science, Tanta University, Egypt

**Keywords:** Lymphocytes, CD62L, IL–2, IL–12, IL–15, IGF–1, T–α1, ATRA, OT–1

## Abstract

**Rationale:** Adoptive T cell therapy depends on the harvesting of the cells from the host, their activation in vitro, and their infusion back to the same host. The way of activating the T cells in vitro is a critical factor for their homing, survival and function in vivo. Sustaining T cell homing molecules, particularly CD62L, is benefic for the trafficking of the adoptive transferred cells.

**Objective:** The aim of the present study is to test whether insulin–like growth factor–1 (IGF–1), thymosin– α1 (T–α1) as well as all–trans retinoid acid (ATRA) alone or in combination with IL–2, IL–12, IL–15 can enhance the activation and survival phenotypes of antigen-activated T cells in vitro.

**Methods & Results:** To this end, OT–1 transgenic T cells were used as a model. These CD8+ T cells recognize OVA peptide presented by MHC class–I. The results showed that antigen stimulation of OT1 cells resulted in their activation as evidenced by the decrease in surface expression of CD62L, analyzed for 3 days after antigen stimulation and was more pronounced on day 5. The addition of IL–12 or IGF–1 alone but not of IL–2, IL–15 augmented OT–1 cell activation measured on day 5. Interestingly, the combination of IL–12 with IGF–1 sustained the expression of CD62L on OT1 cells. Although the addition of ATRA alone or in combination with IL–12 resulted in decreases in CD62L expression on day 3, they showed a dose–dependent effect on the restoration of CD62L expression on day 5. The analysis of the activation–induced cell death (apoptosis) of OT1 cells showed an increased rate of death on day 5 than on day 3–post antigen stimulation. The addition of only IL–12 or IGF–1 alone, but not of IL–2, IL–15 or T– α1, decreased OT1 cell apoptosis on day 3. These anti–apoptotic effects of IL–12 and IGF– 1, however, were recovered on day 5–post stimulation.

**Discussion:** In conclusion, these results indicate that the activation phenotype and the survival of antigen–specific T cells can be differently modulated by immunomodulatory factors, where, interleukin–12 and IGF–1 induced the favorable effect. These results have a significant implication for T cell adoptive immunotherapy in different settings.

## Introduction

Adoptive cell transfer (ACT) represents an important advance in cancer immunotherapy. The success of ACT depends on the functional quality of cells transferred [**1**]. Upon antigen (Ag) recognition, early effector CD8+ T cells are characterized by the expression of high levels of the lymphoid homing molecule CD62L or L–selectin. The expression of CD62L decreases together with the activation progresses, with a re–expression almost restricted to central memory cells [**2–3**]. It is believed that the enhanced anti–Ag activity associated with T cells expressing high levels of CD62L, related to their ability to home the secondary lymph nodes, where effective stimulation can take place [**4**]. Thus, approaches aimed at the generation and maintenance of early effector populations could be valuable in the development of effective ACT–based immunotherapies for cancer. Because cytokines are known to play fundamental roles in the differentiation and maturation of Ag reactive T cells, cytokine conditioning prior to cell transfer has been considered as a means of increasing the proliferation and function of these cells [**5**]. Interleukin–2 and interleukin–15 (IL–2, IL–15) are key participants in T and NK cell activation and function [**6**]. Sharing the beta and gamma–receptor subunits, such as the promotion of T cell proliferation, results in several common functions. On the other hand, due to their distinct alpha–receptor subunits, they also play opposing roles in immune processes such as activation–inducing cell death [**6**].

However, IL–2 and IL–15 could both induce apoptosis and survival; the outcome depends on the cytokine concentration [**7**]. Of significant interest is the role of IL–12. In addition to its role in polarizing Th1 responses and in the maturation of cytotoxic function of NK cells [**8**], IL–12 acts as a third signal along with Ag and IL–2 on naïve CD8+ T cells, increasing the pool of effector and memory cells, two crucial components of a successful ACT approach [**9–10**]. Evidence for this has been provided in recent reports demonstrating increased anti–tumor capabilities of CD8+ T cells after *ex vivo* and *in vitro* culture in the presence of IL–12 [**11–12**].

Increasing evidence indicated that insulin–like growth factor–1 (IGF–1) is involved in the function and development of the immune system. IGF–1 may alter homeostasis in the immune system by modulating lymphocyte generation and survival [**13**].

*In vivo* treatment with IGF–1 enhanced thymic reconstitution in steroid–treated [**14**], aged [**15**], and diabetic [**16**] animals. Hettmer *et al.* [**17**] suggested a role for IGF binding protein as a local growth factor contributing to the proliferation and activation of mononuclear cells. The role of IGF–1 in the regulation of apoptosis has also been suggested both *in vivo* and *in vitro* [**18**]. The production of IGF–1 by thymic epithelial cells [**19**] and the increased IGF–1 receptor expression on T cells after *in vitro* activation with anti–CD3 antibody [**20**] suggested that IGF–1 may play a role in the T cell selection process.

Thymosin–α1 (T–α1), initially isolated from thymus, is now proved to be effective in inhibiting tumoral growth and in controlling infective diseases. Different studies evaluated its immunomodulating effects and showed that T– α1 increased major histocompatibility complex (MHC) class–1, antagonized dexamethasone–induced apoptosis of CD4+CD8+ thymocytes [**21**], primed dendritic cells for antifungal T–helper type 1 resistance through Toll–like receptor signaling [**22**], reduced pancreatic inflammation by regulating differentiation of CD3/CD4+ T cells [**23**] and increased cytokine production. Vitamin A and its metabolite all transretinoic acid (ATRA) have attracted considerable attention as compounds that have a broad range of immune modulating effects on both humoral and cellular immune responses. It was demonstrated that the topical retinoids have significant anti–inflammatory effects in experimental trials [**24**]. While ATRA downregulated the proinflammatory cytokines, the production of immune modulating cytokines was enhanced by ATRA [**25**]. ATRA induced a "priming" of the immune system by increasing the number of T lymphocytes and LPS binding protein expression [**26**], and stimulated T cell proliferation by modulating IL–2–mediated signaling [**27**]. ATRA has been used as monotherapy for treatment of cutaneous T–cell lymphomas for years [**28**]. The combination of ATRA and IFN–gamma could become an efficacious chemoimmunotherapy for the treatment of human glioblastoma [**29**]. ATRA also showed potent effects on hemopoietic stem cell integrity, inhibiting the expansion of human progenitor cells and accelerating their differentiation to B lineage cells [**30**].

The aim of the present study was to define the immunomodulatory factors that can increase survival and sustain CD62L expression in antigen–specific T cells. To this end, the effects of IL–2, IL–12, IL–15, IGF–1, T– α1 as well as ATRA alone or in combination were tested *in vitro* utilizing OT1 transgenic T cells as a model.

## Materials and methods

**Mice:** OT–1 T cell receptor (TCR) transgenic mice on C57Bl/6 (B6) background were purchased from Jackson Laboratory (Bar Harbor, ME). OT–1 mice were bred with B6.SJL mice to generate Ly5.1+/Ly5.1+ mice heterozygous for the OT–1 TCR (Vα2/Vα5) transgene confirmed by flow cytometry with monoclonal antibody (mAb) specific for Vα2. All animals were housed under specific pathogen–free conditions in accordance with institutional and federal guidelines at the Medical University of South Carolina, USA.

**Antibodies and reagents:** Anti–CD16/CD32, and FITC–, PE–, APC–, and cychrome–conjugated monoclonal antibodies (mAbs) were purchased from Pharmingen, San Diego, CA. The major histocompatability (MHC) class–I SIINFEKL OVA peptide (OVAp) (American Peptide Company, Inc., Sunnyvale, CA) was dissolved in 10% DMSO (Sigma, St. Louis, MO) and diluted in phosphate buffered solution (PBS). Cytokines IL–2, IL–12, and IL–15 (Systems, Minneopolis, MN) were stored as a lyophilized powder at –20°C, and reconstituted immediately prior to use in 0.1% bovine serum albumin (BSA) in PBS. IGF–1, T–α1, and ATRA were purchased from Sigma, San Diego, CA.

A**ntigen–induced T cell activation:** OT–1 cells were prepared and cultured as previously described by Salem et al. [**31**]. In brief, one or 2 OT–1 mice were sacrificed and spleens and peripheral (cervical, auxiliary, brachial, and inguinal) and mesenteric lymph nodes were harvested and pooled. Single cell suspension was prepared in PBS by grinding these tissues between the 2 rough ends of microscopic slides. Cells were washed twice and re–suspended in RPMI medium. Unfractionated OT–1 cells (1 × 105) were cultured in triplicate in 6–well plate for 3 days in RPMI medium containing 1 μg/ml OVAp and the tested growth factors at the dose indicated in each experiment. Cells were harvested and washed twice and were freshly processed for the activation phenotype and apoptosis measured by flow cytometry.

**Measuring T cell activation by flow cytometry:** Staining for flow cytometry was performed as previously described by Salem et al. [**31**]. Fresh cells (0.5–1 × 106) were treated with anti–CD16/CD32 for 5 minutes on ice and then stained with the indicated conjugated mAbs, and incubated for 30 min on ice. The cells were washed twice and resuspended in 0.3 ml of 0.5% BSA, 0.02% sodium azide solution. Cells were washed, and analyzed by flow cytometry using the Cell Quest software package (Becton Dickinson, San Jose, CA).

**Measuring apoptosis by flow cytometry:** To measure apoptosis, cells were stained for the surface markers as described above and cells were then washed twice with FCAS buffer and twice with Annexin–V binding buffer (Pharmingen). Cells were incubated at room temperature for 15 minutes and then washed and analyzed immediately by flow cytometry.

## Results

**Fig. 1:**
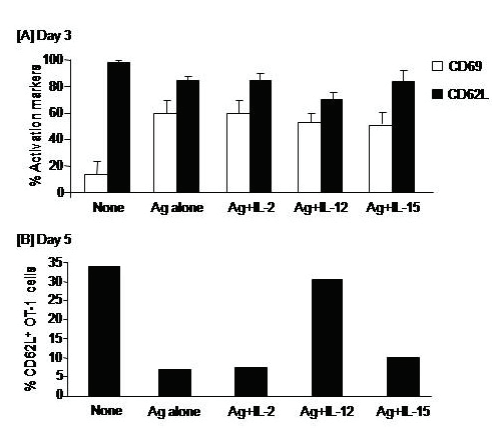
Impacts of cytokines on the antigen-specific activation of CD8+ T cells. OT–1 (CD8+) T cells were harvested from spleen and LN of naïve TCR transgenic mice and 1 × 106 cells were cultured in RPMI medium and stimulated with the MHC class–I peptide (SIINFEKL) from OVA albumin in the presence or absence of 10 ng/ml of interleukin (IL)–2, IL–12, or IL–15 cytokines. Cells were harvested on day 3 to assay expression of the early activation receptors CD69 and CD62L (A) and on day 5 to analyze the expression of only CD62L (B).

CD8+ T cells stimulated with Ag showed higher levels of CD69 and lower levels of CD62L expression, compared to the CD69 and CD62L expression on unstimulated CD8+ T cells, on day 3 indicating their simulation. Of Note, the decrease in CD62L expression after Ag stimulation was further augmented on day 5 post–stimulation. Addition of IL–2, IL–12 or IL– 15 to CD8+ T cells culture during Ag stimulation did not alter the phenotypic activation of CD8+ T cells when cells were harvested on day 3. The expression of CD62L assessed on CD8+ T cells on day 5 was much lower than on day 3. However, only CD8+ T cells that had conditioned with IL–12 showed higher levels of CD62L (**Fig. 1**).

**Fig. 2:**
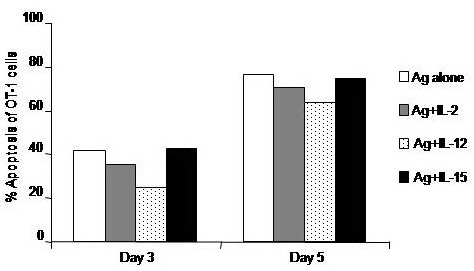
Impacts of cytokines on the activation induced cell death of antigen–stimulated CD8+ T cells. OT–1 (CD8+) T cells were harvested from spleen and LN of naïve TCR transgenic mice and 1 × 106 cells were cultured in RPMI medium and stimulated with the MHC class–I peptide SIINFEKL from OVA albumin in the presence or absence of 10 ng/ml of IL–2, IL–12, or IL–15 cytokines. Cells were harvested on days 3 and 5 and their early apoptosis was analyzed by annexin–v assay.

Addition of IGF–1 alone or in combination with IL–2, IL–12 or IL–15 to CD8+ T cells culture during Ag stimulation did not significantly alter the expression of either CD69 or CD62L on CD8+ T cells on day 3. The expression of CD62L assessed on CD8+ T cells on day 5 was much lower than on day 3. However, only CD8+ T cells that had been conditioned with combined IGF–1 and IL–12 showed higher levels of CD62L expression. The magnitude of this increase reached about 6–folds of the corresponding values of Ag stimulated CD8+ T cells (**Fig. 2**).

**Fig. 3:**
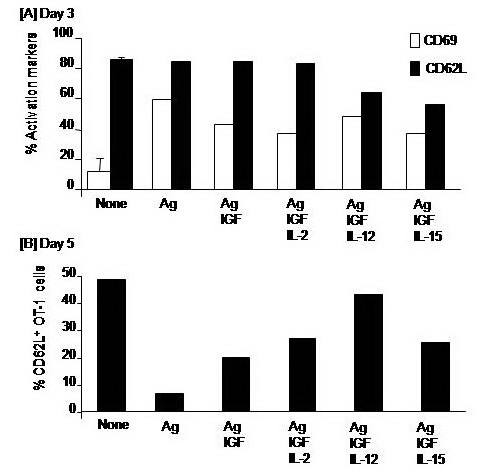
Impacts of a combinatorial treatment with IGF and cytokines on the antigen–specific activation of CD8+ T cells were harvested from spleen and LN of naïve TCR transgenic mice and 1 × 106 cells were cultured in RPMI medium and stimulated with the MHC class–I peptide SIINFEKL from OVA albumin in the presence or absence of 10ng/ml insulin growth factor (IGF). Then, cells stimulated with IGF were further conditioned with either 10ng/ml IL–2, IL–12, or IL–15 cytokines. Cells were harvested on day 3 to assay expression of the early activation receptors CD62L and CD69 (A) and on day 5 to analyze the expression of only CD62L (B).

Addition of T–α1 alone or in combination with IL–2, IL–12 or IL–15 to CD8+ T cells culture during Ag stimulation did not alter the expression of either CD69 or CD62L on CD8+ T cells on day 3. The expression of CD62L assessed on CD8+ T cells on day 5 was much lower than on day 3. However, only CD8+ T cells that had been conditioned with T–α1 and IL–12 in combination showed higher levels of CD62L expression. The magnitude of this increase reached about 3–folds of the corresponding values of Ag–stimulated CD8+ T cells (**Fig. 3**).

**Fig. 4:**
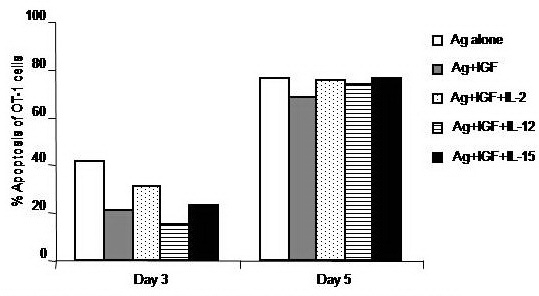
Impacts of a combinational treatment with IGF and cytokines on the antigen-specific activation of CD8+ T cells. OT–1 (CD8+) T cells were harvested from spleen and LN of naïve TCR transgenic mice and 1 x 106 cells were cultured in RPMI medium and stimulated with the MHC class–I peptide SIINFEKL from OVA albumin in the presence or absence of 10ng/ml insulin growth factor (IGF). Cells stimulated with IGF were also conditioned with either 10 ng/ml IL–2, IL–12, or IL–15 cytokines. Cells were harvested on days 3 and 5 and their early apoptosis was analyzed by annexin–v assay.

Addition of ATRA with doses 1, 5, 10 or 15 μM to CD8+ T cells culture during Ag stimulation did not alter the expression of CD62L on CD8+ T cells on day 3. The expression of CD62L assessed on CD8+ T cells on day 5 was much lower than on day 3. However, only CD8+ T cells that had been conditioned with ATRA with doses 10 and 15 μM showed higher levels of CD62L expression. The magnitude of this increase reached about 2.8– and 3–folds of the corresponding values of Ag–stimulated CD8+ T cells, respectively (**Fig. 4**).

**Fig. 5:**
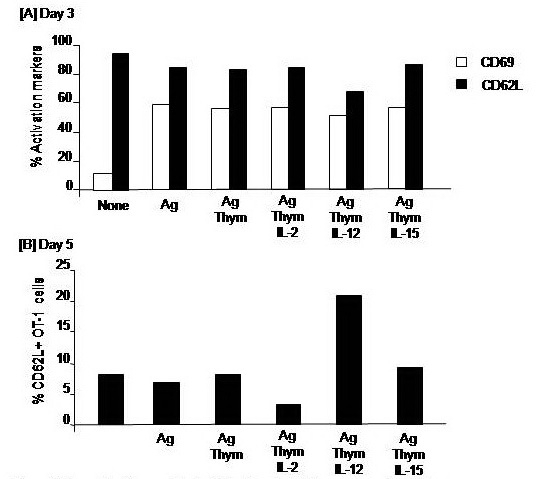
Impacts of a combinatorial treatment with thymosin and cytokines on the antigen–specific activation of CD8+ T cells. OT–1 (CD8+) T cells were harvested from spleen and LN of naïve TCR transgenic mice and 1 × 106 cells were cultured in RPMI medium and stimulated with the MHC class–I peptide SIINFEKL from OVA albumin in the presence or absence of 10 ng/ml thymosin (Thym). Cells stimulated with Thym were also conditioned with either 10 ng/ml IL–2, IL–12, or IL–15 cytokines. Cells were harvested on day 3 to assay expression of the early activation receptors CD62L and CD69 (A) and on day 5 to analyze the expression of only CD62L (B).

Addition of ATRA with doses 1, 5, 10 or 15 μM in combination with IL–12 to CD8+ T cells culture during Ag stimulation did not alter the expression of CD62L on CD8+ T cells on day 3. The expression of CD62L assessed on CD8+ T cells on day 5 was much lower than on day 3. However, only CD8+ T cells that had been conditioned with ATRA with doses 5, 10 and 15 μM showed higher levels of CD62L expression. The magnitude of this increase reached about 2.7–, 3– and 3.7–folds of the corresponding values of Ag–stimulated CD8+ T cells, respectively (**Fig. 5**).

**Fig. 6:**
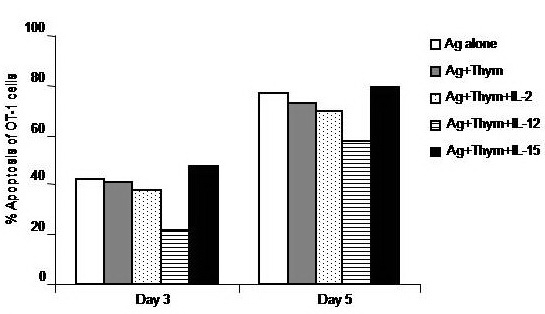
Impacts of a combinational treatment with Thym and cytokines on the antigen–specific activation of CD8+ T cells. OT–1 (CD8+) T cells were harvested from spleen and LN of naïve TCR transgenic mice and 1 × 106 cells were cultured in RPMI medium and stimulated with the MHC class–I peptide SIINFEKL from OVA albumin in the presence or absence of 10 ng/ml thymosin (Thym). Cells stimulated with Thym were also conditioned with either 10 ng/ml, IL–2, IL–12, or IL–15 cytokines. Cells were harvested on days 3 and 5 and their early apoptosis was analyzed by annexin–v assay.

To test whether conditioning CD8+ T cells with IL–2, IL–12 or IL–15 impacts on Ag–induced T cells apoptosis, expression of Annexin V, as a marker of apoptosis, was measured by flow cytometry. It was found that stimulation of cells with Ag alone induced about 40% of the cultured cells to undergo apoptosis on day 3, which further augmented on day 5. Treatment of CD8+ T cells with only IL–12, but not IL–2 or IL–15 decreased the apoptosis level of CD8+ T cells by about 41% of the corresponding value of Ag–stimulated CD8+ T cells when measured on day 3. This anti–apoptotic effect of IL–12, however, was lost by day 5 (**Fig. 6**).

**Fig. 7:**
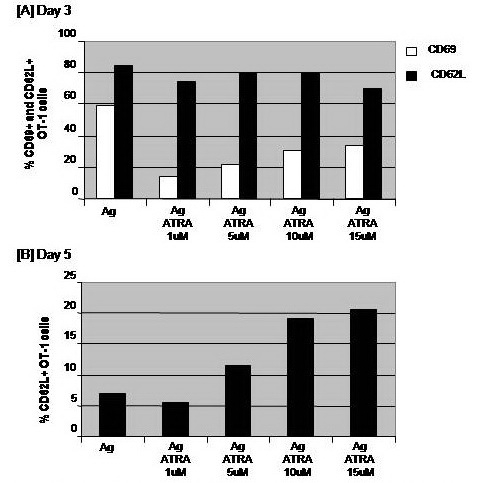
Impacts of treatment with different concentration of ATRA on the antigen-specific activation of CD8+ T cells. OT–1 (CD8+) T cells were harvested from spleen and LN of naïve TCR transgenic mice and 1 × 106 cells were cultured in RPMI medium and stimulated with the MHC class–I peptide SIINFEKL from OVA albumin in the presence or absence of the indicated concentrations of ATRA. Cells were harvested on day 3 to assay expression of the early activation receptors CD62L and CD69 (A) and on day 5 to analyze the expression of only CD62L (B).

Treatment of CD8+ T cells with IGF–1 alone or in combination with IL–12 or IL–15, but not IL–2, decreased the percentage of apoptosis of these cells by about 51.1 and 62.9% of the corresponding value of Ag–stimulated CD8+ T cells, respectively, when measured on day 3. However, the anti–apoptotic effect of IGF–1 alone or in combination with IL–12 or IL–15 was less pronounced on day 5 (**Fig. 7**).

**Fig. 8:**
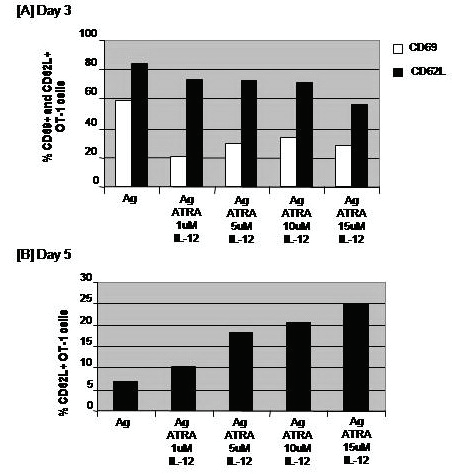
Impacts of a combinatorial treatment with ATRA and IL–12 on the antigen–specific activation of CD8+ T cells. OT–1 (CD8+) T cells were harvested from spleen and LN of naïve TCR transgenic mice and 1 × 106 cells were cultured in RPMI medium and stimulated with the MHC class–I peptide SIINFEKL from OVA albumin in the presence or absence of different concentration of ATRA. Cells stimulated with ATRA were concomitantly treated with 10 ng/ml IL–12. Cells were harvested on day 3 to assay expression of the early activation receptors CD62L and CD69 (A) and on day 5 to analyze the expression of only CD62L (B).

Combined treatment of CD8+ T cells with only T–α1 and IL–12, but not T–α1 alone or in combination with IL–2 or IL–15, decreased the percentage of apoptosis of these cells by about 47.5% of the corresponding value of Agstimulated CD8+ T cells when measured on day 3. However, the anti–apoptotic effect of a combined treatment with T–α1 and IL–12 was less pronounced by day 5 (**Fig. 8**).

**Fig. 9:**
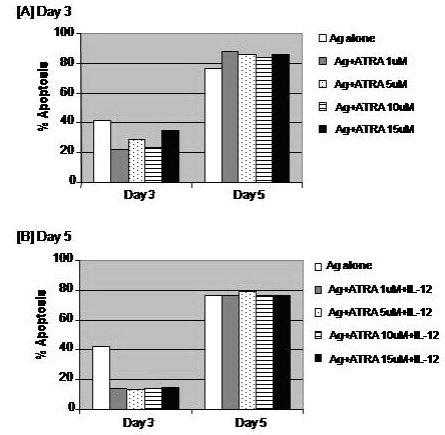
Impacts of treatment with ATRA alone or in combinational with IL–12 on the antigen–specific activation of CD8+ T cells. OT–1 (CD8+) T cells were harvested from spleen and LN of naïve TCR transgenic mice and 1 × 106 cells were cultured in RPMI medium and stimulated with the MHC class–I peptide SIINFEKL from OVA albumin in the presence or absence of different concentration of ATRA (A). A separate set of ATRA–treated cells were concomitantly treated with 10 ng/ml IL–12 (B). Cells were harvested on day 5 post treatment and their apoptosis was determined by annexin–v binding assay.

Treatment of CD8+ T cells with ATRA (1 and 10 μM), but not 5 or 15 μg, decreased the percentage of apoptosis of these cells by about 49.2 and 44.7% of the corresponding value of Ag#x2013;stimulated CD8+ T cells, respectively when measured on day 3. However, the anti–apoptotic effect of ATRA treatment with doses 1 and 10 μg was abrogated by day 5 (**Fig. 9a**). Combined treatment of CD8+ T cells with ATRA (1, 5, 10 and 15 μM) and IL–12 decreased the percentage of apoptosis of these cells by about 67.0, 70.1, 66.5 and 65.8% of the corresponding value of Ag–stimulated CD8+ T cells, respectively when measured on day 3. However, the anti–apoptotic effect of a combined treatment of ATRA with doses 1, 5, 10 and 15 μM and IL–12 was recovered by day 5 (**Fig. 9b**).

## Discussion

CD8+ T cells are a critical component of the cellular immune response. They play an important role in the control of viral infection and eliminating cells with malignant potential [**32–33**]. However, attempts to generate and expand human CD8+ T cells *in vitro* for an adoptive immunotherapy had been conducted with limitation of the very low frequency of CD8+ T cells in blood. Therefore, several expansion protocols were developed to obtain large and efficient numbers of human CD8+ T cells for use in adoptive immunotherapies [**34**]. In this study IL–12 alone, but not IL–2 nor IL–15, as well as IGF–1 alone augmented the activation of CD8+ OT1 T cells. Interestingly, combination of IL –12 with IGF–1 sustained the expression of CD62L on OT1 cells. Analysis of the activation–induced cell death (apoptosis) of OT1 cells showed that addition of only IL–12 or IGF–1 alone, but not IL–2, IL–15 or T–α1, to Ag–stimulated CD8+ T cells decreased the percentage of apoptosis of these cells.

*In vitro* or *in vivo* treatment with IL–12 resulted in enhanced activity of T and NK cells which is characterized by increased secretion of IFN–γ [**31**]. Such enhanced immune response is, in part, the result of the direct stimulation of antigen presenting cells. A previous study by Salem *et al.* [**35**] showed that paracrine administration of IL–12 enhanced the functional capability of spleen dendritic cells to present peptide to naïve CD8+ T cells. IL–12 can also directly affect the development of CD8+ T cell–mediated responses [**32**]. By signaling directly through its receptor on CD8 T cells, IL–12 influences their differentiation to favor the generation of fully activated effectors [**34**]. It is known that optimal clonal expansion and acquisition of effector function requires a third signal that can be provided by IL–12 and that, in the absence of third signal, tolerance can occur [**9–10**]. In support of these findings, Chang *et al.* [**36**] reported that IL–12 priming at the time of antigenic stimulation increased the primary expansion of CD8+ T cells by reducing cell death, rather that by increasing cell proliferation, resulting in a larger CD8+ T cell memory pool. The ability of IL–12 to enhance proliferation of activated CD8+ T cells has been associated with enhanced expression of IL–2Rα in response to IL–12R engagement [**10**]. IL–12R expression is upregulated upon T cell receptor (TCR) activation [**37**], however low levels of expression were reported in resting NK cells, dendritic cells and B cell lines [**38–39**]. Historically, the expression of IL–12R was confined to activated Th1 cells [**40–41**], however the pivotal role of IL–12 in providing third signal for the clonal expansion of naïve CD8+ T cells, demonstrates a similar pattern of expression on activated CD8+ T cells [**10**].

IGF–1 has been implicated to play a regulatory role in T cell development and in T cell function [**42**]. *In vivo* activated T lymphocytes exhibit altered expression of IGF– 1 receptor compared to the naïve T lymphocyte pool [**43**]. IGF–1 receptors were readily detectable on a wide variety of the immune cells, including T cells, B cells and monocytes, but the binding capacity for IGF–1 was monocytes, B cells and T cells. The level of IGF–1 receptor expression was increased several–fold after stimulation of both CD4+ and CD8+ T–cell subsets. IGF–1 is also expressed in many tumor cell lines and had a role in both normal cell proliferation and in the growth of cancers. Several approaches to target IGF–1 signaling resulted in the inhibition of the growth of a broad range of tumor cells [**44**]. IGF–1 was shown to protect cells from apoptosis by different stimuli and conditions [**45**]. It may alter homeostasis in the immune system by modulating lymphocyte generation and survival [**13**].

Mice deficient in dendritic epidermal T cells had a notable increase in epidermal apoptosis that was abrogated by the addition of IGF–1 [**46**]. T cell death–associated gene 51 plays an important role in the anti–apoptotic effects of IGF–I. Thus, effective strategies to generate competent CD8+ T cells for adoptive transfer should promote not only the generation of key subpopulations with superior anti–tumor activity, but also the maintenance of their viability *in vivo*. The present results suggest that antigen priming in the presence of both IL– 12 and IGF–1 accomplishes both ends. It not only directly modulates the expression of CD62L on activated CD8+ T cells, but also selectively promotes the survival of activated CD8+ T cells expressing high levels of CD62L. In conclusion, the present results indicate that the activation phenotype and the survival of antigen–specific T cells can be differentially modulated by different immunomodulatory factors, where, interleukin– 12 and IGF–1 induced the favorable effect. These results have a significant implication for T cell adoptive immunotherapy in different settings.

## References

[1] Gattinoni L, Powell DJ Jr, Rosenberg SA, Restifo NP (2006). Adoptive immunotherapy for cancer: building on success. Nat. Rev. Immunol.

[2] Jung TM, Gallatin WM, Weissman IL, Dailey MO (1988). Down-regulation of homing receptors after T cell activation.. J. Immunol.

[3] Sallusto F, Lenig D, Forster R (1999). Two subsets of memory T lymphocytes with distinct homing potentials and effector functions.. Nature.

[4] Springer TA (1994). Traffic signals for lymphocyte recirculation and leukocyte emigration: the multistep paradigm. Cell.

[5] Schluns KS, Lefrancois L (2003). Cytokine control of memory T-cell development and survival. Nat. Rev. Immunol..

[6] Lin JX, Spolski R, Leonard WJ (2008). Critical role for Rsk2 in T-lymphocyte activation. Blood.

[7] Zambricki E, Shigeoka A, Kishimoto H (2005). Signaling T-cell survival and death by IL-2 and IL-15. Am. J. Transplant.

[8] Watford WT, Moriguchi M, Morinobu A, O'Shea JJ (2003). The biology of IL-12: coordinating innate and adaptive immune responses. Cytokine Growth Factor Rev.

[9] Curtsinger JM, Johnson CM, Mescher MF (2003). CD8 T cell clonal expansion and development of effector function require prolonged exposure to antigen, costimulation and signal 3 cytokine. J. Immunol.

[10] Valenzuela JO, Hammerbeck CD, Mescher MF (2005). Cutting edge: Bcl-3 up-regulation by signal 3 cytokine (IL-12) prolongs survival of antigenactivated CD8 T cells. J. Immunol.

[11] Macgregor JN, Li Q, Chang AE (2006). Ex vivo culture with interleukin (IL)-12 improves CD8 T-cell adoptive immunotherapy for murine leukemia independent of IL-18 or IFN-gamma but requires perforin.. Cancer Res.

[12] Diaz-Montero MC, El-Naggar S, El-Khami A (2007). Priming of naïve CD8 T cells in the presence of IL-12 selectively enhances the survival of CD8 CD62L cells and results in superior anti-tumor activity in a tolerogenic murine model. Cancer Immunol. Immunother.

[13] Hinton PS, Peterson CA, Dahly EM, Ney DM (1998). IGF-I alters lymphocyte survival and regeneration in thymus and spleen after dexamethasone treatment. Am. J. Physiol.

[14] Hinton PS, Peterson CA, Lo HC (1995). Insulin-like growth factor-I enhances immune response in dexamethasone-treated or surgically stressed rats maintained with total parenteral nutrition. JPEN J. Parenter. Nutr.

[15] Campbell DJ, Rawlings JM, Heaton PR (2004). Insulin-like growth factor-I (IGF-I) and its association with lymphocyte homeostasis in the ageing cat.. Mech.Ageing Dev.

[16] Geenen V, Brilot F, Louis C (2005). Importance of a thymus dysfunction in the pathophysiology of type 1 diabetes. Rev. Med. Liege.

[17] Hettmer S, Dannecker L, Foell J (2005). Effects of insulin-like growth factors and insulin-like growth factor binding protein-2 on the in vitro proliferation of peripheral blood mononuclear cells. Hum. Immunol.

[18] Han X, Amar S (2003). IGF-1 signaling enhances cell survival in periodontal ligament fibroblasts vs. gingival fibroblasts. J. Dent. Res.

[19] Timsit J, Savino W, Safieh B (1992). Growth hormone and insulin-like growth factor-I stimulate hormonal function and proliferation of thymic epithelial cells. J. Clin. Endocrinol. Metab.

[20] Johnson EW, Jones LA, Kozak RW (1992). Expression and function of insulin-like growth factor recepors on anti-CD3-activated human T lymphocytes.. J. Immunol.

[21] Baumann CA, Badamchian M, Goldstein AL (2007). Thymosin-α1 is a time and dose-dependent antagonist of dexamethasone-induced. Int. J. Immunopharmacol.

[22] Romani L, Bistoni F, Perruccio K (2006). Thymosin-α1 activates dendritic cell tryptophan catabolism and establishes a regulatory environment for balance of inflammation and tolerance.. Blood.

[23] Yao W, Zhu Q, Yuan Y (2007). Thymosin-α1 improves severe acute pancreatitis in rats via regulation of peripheral T cell number and cytokine serum level. J. Gastroenterol. Hepatol.

[24] Wolf JE Jr (2002). Potential antiinflammatory effects of topical retinoids and retinoid analogues. Adv. Ther.

[25] Wang X, Allen C, Ballow M (2007). Retinoic acid enhances the production of IL-10 while reducing the synthesis of IL-12 and TNFalpha from LPS-stimulated monocytes/macrophages. J. Clin.Immunol.

[26] Seguin-Devaux C, Hanriot D, Dailloux M (2005). Retinoic acid amplifies the host immune response to LPS through increased T lymphocytes number and LPS binding protein expression.. Mol. Cell. Endocrinol.

[27] Engedal N, Gjevik T, Blomhoff R, Blomhoff HK (2006). All-trans retinoic acid stimulates IL-2-mediated proliferation of human T lymphocytes: early induction of cyclin D3. J. Immunol.

[28] Zhang C, Duvic M (2006). Treatment of cutaneous T- cell lymphoma with retinoids. Dermatol. Ther.

[29] Haque A, Banik NL, Ray SK (2007). Emerging role of combination of alltrans retinoic acid and interferongamma as chemoimmunotherapy in the management of human glioblastoma. Neurochem. Res.

[30] Chen X, Esplin BL, Garrett KP (2008). Retinoids accelerate B lineage lymphoid differentiation. J. Immunol.

[31] Salem ML, Gillanders WE, Kadima AN (2006). Review: novel nonviral delivery approaches for interleukin-12 protein and gene systems: curbing toxicity and enhancing adjuvant activity. J. Interferon Cytokine Res.

[32] Al-Shanti N, Aldahoudi Z (2007). Human purified CD8 T cells: Ex vivo expansion model to generate a maximum yield of functional cytotoxic cells. Immunol. Invest.

[33] Wood AH, Zhang X, Farber DL, Strome SE (2007). CD8 memory T lymphocytes from bone marrow: Immune function and therapeutic potential. Crit Rev. Immunol.

[34] Pearce EL, Shen H (2007). Generation of CD8 T cell memory is regulated by IL-12. J. Immunol.

[35] Salem ML, Kadima AN, Zhou Y (2004). Paracrine release of IL-12 stimulates IFNgamma production and dramatically enhances the antigen-specific T cell response after vaccination with a novel peptide-based cancer vaccine. J Immunol.

[36] Chang J, Cho JH, Lee SW (2004). IL-12 priming during in vitro antigenic stimulation changes properties of CD8 T cells and increases generation of effector and memory cells. J. Immunol.

[37] Gately MK, Renzetti LM, Magram J (2008). Annu. Rev. Immunol.

[38] Grohmann U, Belladonna ML, Bianchi R (1998). IL-12 acts directly on DC to promote nuclear localization of NF-kappaB and primes DC for IL-12 production. Immunity.

[39] Adorini L, Gubler U, Presky DH (1998). The interleukin-12/interleukin-12-receptor system: role in normal and pathologic immune responses. Annu Rev Immunol.

[40] Rogge L, Barberis-Maino L, Biffi M (2004). TDAG51 mediates the effects of insulin-like growth factor I (IGF-I) on cell survival. J. Biol. Chem.

[41] Szabo SJ, Dighe AS, Gubler U, Murphy KM (1997). Regulation of the interleukin (IL)-12R beta 2 subunit expression in developing T helper 1 (Th1) and Th2 cells. J. Exp. Med.

[42] Kooijman RK, Scholtens LE, Rijkers GT, Zegers BJ (1995). Differential expression of type I insulin- like growth factor receptors in different stages of human T cells. Eur. J. Immunol.

[43] Omazic B, Nasman-Bjork I, Permert J, Lundkvist I (2007). Insulin-like growth factor-1 receptor RNA expression in hematopoietic stem cell transplanted patients does not correlate with graft-versus-host disease. Immunol. Invest.

[44] Conti L, Regis G, Longo A (2007). In the absence of IGF-1 signaling, IFN-gamma suppresses human malignant T-cell growth. Blood.

[45] Di Marzio L, Moretti S, D’Alo S (1999). Acetyl-L-carnitine administration increases insulin-like growth factor 1 levels in asymptomatic HIV-1- infected subjects: correlation with its suppressive effect on lymphocyte apoptosis and ceramide generation. Clin. Immunol.

[46] Sharp IL, Jameson JM, Cauvi G, Havran WL (2005). Dendritic epidermal T cells regulate skin homeostasis through local production of insulinlike growth factor 1. Nat. Immunol.

